# The role of microbiota in the chronic prostatitis/chronic pelvis pain syndrome: a review

**DOI:** 10.3389/fmicb.2025.1488732

**Published:** 2025-03-12

**Authors:** An-Qi Deng, Shao-Yu Yue, Di Niu, Dan-Dan Zhang, Bing-Bing Hou, Li Zhang, Chao-Zhao Liang, He-Xi Du

**Affiliations:** ^1^Department of Urology, The First Affiliated Hospital of Anhui Medical University, Anhui Medical University, Hefei, Anhui, China; ^2^Institute of Urology, Anhui Medical University, Hefei, Anhui, China; ^3^Anhui Province Key Laboratory of Urological and Andrological Diseases Research and Medical Transformation, Hefei, Anhui, China; ^4^The Second Clinical Medical School, Anhui Medical University, Hefei, Anhui, China; ^5^Clinical College of Anhui Medical University, Hefei, Anhui, China

**Keywords:** microbiota, CP/CPPS, pathogenesis, treatment microbiota, treatment

## Abstract

Chronic prostatitis/Chronic pelvis pain syndrome (CP/CPPS), a kind of frequent urinary condition among adult males, has caused a lot of inconvenience to patients in life, whose pathogenesis is unclear. Current evidence suggests that it is most likely to be an autoimmune disease. Symbiotic microbes, a highly diverse biological community that harbors trillions of microbes in each region of the human body, have gradually made people realize their important role in immune regulation, material metabolism, and health maintenance. In recent years, increasing studies have shown a connection between microbiota and CP/CPPS. In view of this, we performed this review to summarize the literature pertaining to microbiota and its association with the pathophysiological mechanism of CP/CPPS. In addition, we gleaned the latest progress in the therapeutic strategy of CP/CPPS that related to microbiota regulation in order to offer new perspectives on the management of CP/CPPS.

## 1 Introduction

Chronic prostatitis/chronic pelvic pain syndrome (CP/CPPS), a prevalent urological disorder, demonstrates a substantial incidence rate reaching 8.2% (Krieger et al., 2008). This condition not only poses significant health risks to affected individuals but also imposes substantial socioeconomic burdens (Bartoletti et al., 2007; Suskind et al., 2013). According to the National Institutes of Health (NIH) classification system, prostatitis is categorized into four distinct types, among which CP/CPPS (NIH category III) accounts for over 90% of clinical cases (Rees et al., 2015). The hallmark symptom of CP/CPPS manifests as recurrent and persistent pelvic pain, typically enduring for more than 3 months, frequently accompanied by varying degrees of lower urinary tract symptoms and sexual dysfunction. Characteristically, bacterial cultures from expressed prostatic secretions (EPS), semen samples, or post-prostatic massage urine specimens (VB3) typically yield negative results, rendering CP/CPPS diagnosis particularly challenging and its underlying etiology a subject of ongoing debate (Habermacher et al., 2006; Schaeffer et al., 2002). Emerging evidence, however, increasingly supports the hypothesis that CP/CPPS may represent an autoimmune-mediated condition (Lu et al., 2018).

The human microbiota, constituting a complex ecosystem of microorganisms established at birth, coexists with the host as an essential biological component and undergoes parallel development throughout the host’s lifespan (Dominguez-Bello et al., 2019). From birth to death, dynamic alterations take place spanning the development trajectory of human microbiota and giving rise to the shaping of phenotypes (Ochman et al., 2010). Ascribing to the tremendous reproduction rates compared to humans and the exceptional adaptability of the metagenome to environmental changes, microbiota may play a crucial role in maintaining systemic homeostasis across multiple host systems (Levy et al., 2017). Consequently, microbial communities maintain a symbiotic relationship with host health, while microbial dysbiosis can readily contribute to disease pathogenesis. Tracing back to 2008, the NIH Common Fund Office initiated the Human Microbiome Project (HMP), a landmark endeavor designed to uncover the fundamental relationships between human microbiota and health, thereby highlighting the critical role of microbial communities in human physiology (Integrative HMP (iHMP) Research Network Consortium, 2019).

Over the past few years, investigators have gradually revealed the inseparable association between human microbiota and various diseases (Ruff et al., 2020; Young, 2017). It is noteworthy that microbiota actively engages with the immune system, potentially to be linked to numerous immune-mediated disorders. Scientific evidence demonstrates that an alteration in certain bacteria in the gut may induce the development of rheumatoid arthritis (RA) (Scher et al., 2013; Vaahtovuo et al., 2008). In another research, Zhang et al. (2023) freshly published their study highlighting that gut microbiota influences autoimmune thyroiditis pathogenesis through hydrogen sulfide (H_2_S) regulation. Collectively, these findings establish microbial involvement in diverse immunological disease processes.

**FIGURE 1:**
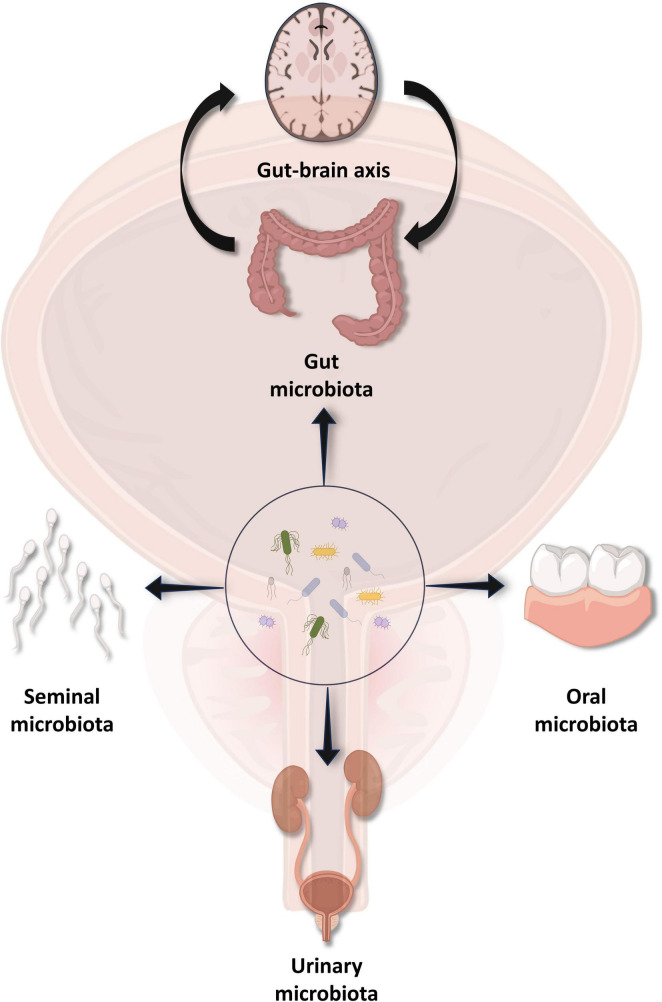
Various microbiota of different regions and chronic prostatitis/chronic pelvis pain syndrome (CP/CPPS). Microbiota present in the gut, oral cavity, urinary system, and semen have proven to correlate with etiopathogenesis as well as the treatment of CP/CPPS.

Similar to their involvement in immunological disease pathogenesis, the disturbance of human microbiota also plays a modulating role in prostatic diseases (Porter et al., 2018). What caught our attention was that related studies on CP/CPPS over the past few years have collectively suggested that the microbiota may be able to address the pending pathogenesis of CP/CPPS (Choi et al., 2020; Wu et al., 2020). Some microbiota-cenric interventions emerged as the key crucial elements in prostate biology, which are paramount for establishing prevention and developing therapeutic strategies (Terrisse et al., 2022; Uchugonova et al., 2015). Despite growing evidence linking microbiota to CP/CPPS, the precise mechanisms underlying this interaction remain largely undefined. Based on such conditions, this review aims to: systematically evaluate current evidence regarding microbial involvement in CP/CPPS pathogenesis, with particular emphasis on disease etiology and progression (Figure 1 and Table 1); and (b) critically assess recent advances in both clinical and basic research on CP/CPPS (Table 2), thereby providing a foundation for well-designed studies and innovative therapeutic approaches.

**TABLE 1:** Overviewing of select studies investigating the various categories of microbiota in chronic prostatitis/chronic pelvis pain syndrome (CP/CPPS).

Author (year)	Country	Study design	Sample information	Sample type	Main outcomes	References
**Gut microbiota**						
Shoskes 2016	United States	Case-control	25 CP/CPPS, 25 controls	Rectal exam	Lower α-diversity was detected in CP/CPPS versus controls, significantly in those whose symptom duration > 48 months. CP/CPPS patients have relatively less abundant *Prevotella* in the guts.	Shoskes et al., 2016b
Konkol 2019	Finland	Animal experiment	–	Fecal samples	A lower abundance of *Bacteroides uniformis*, *Lactobacillus*, and *Lachnospiraceae* was detected in CNP mice, and a higher abundance of *Rikenellaceae*, *Odoribacter*, *Clostridiaceae*, *Allobaculum* and *Peptococcaceae* in healthy mice. Treatment with the GGM extract significantly reduced the abundance of *Odoribacter* and *Clostridiaceae* levels and increased the *Bacteroides uniformis* levels.	Konkol et al., 2019
Du 2020	China	Animal experiment	–	Fecal samples	Altered gut microbiota induced depressive-like symptoms in EAP mice. The α-diversity and β-diversity of gut microbiota were altered in EAP mice. The relative abundance of microbiota in the EAP group has been altered in all six levels (phylum, class, order, family, genus, and species) compared to the control group. Fecal microbiota transplantation significantly altered the composition of gut microbiota in pseudo-germ-free mice. Gut microbiota from the control group and EAP group alleviated and induced depressive symptoms, respectively.	Du et al., 2020
Du 2022	China	Animal experiment	–	Fecal samples	The α-diversity gut microbiota was altered in EAP mice. The relative abundance of microbiota in the EAP group has been altered in all six levels (phylum, class, order, etc.). At the phylum level, *Firmicutes*, *Nitrospirae*, *Gemmatimonadetes*, and *Fusobacteria* were decreased and the *Bacteroidetes*, *Cyanobacteria*, and *Patescibacteria* were increased significantly. At the genus level, *Lactobacillus* was decreased and the other nine were significantly decreased. Altered gut microbial composition reduced propionic acid production which in turn leads to an imbalance in Th17/Treg cell differentiation and ultimately plays an important role in mediating CP/CPPS disorders.	Du et al., 2022
Wang 2023	China	Case-control	41 CP/CPPS, 43 controls	Fecal samples	No significant differences in α-diversity, but significant differences in the relative abundance of several bacterial genera. β-diversity revealed significant differences between the two groups. Three bacteria were highly representative (be highly representative) and seven bacteria were underrepresented in the CP/CPPS group compared to the control group.	Wang S. et al., 2023
Du 2024	China	Animal experiment	–	Fecal samples	Alcohol mediated changes in gut microbiota and increased intestinal permeability. Alcohol intake adjusted the proportion of Th17 cells and associated inflammatory factor levels upwardly. Alcohol mediated gut microbiota-associated cholesterol metabolism pathways that exacerbated CP/CPPS symptoms.	Du et al., 2024
**Oral microbiota**						
Joshi 2010	United States	Cross-sectional	35 subjects with abnormal findings on digital rectal examination or elevated PSA	PSA	Mean PSA levels were significantly higher in subjects with moderate/severe prostate inflammation than in subjects with none/mild inflammation. Subjects with moderate/severe prostatitis and Poorer periodontal tissue status have a higher PSA level.	Joshi et al., 2010
Nabil 2015	United States	Cross-sectional	27 subjects with abnormal findings on digital rectal examination or elevated PSA	PSA	Periodontal treatment reduced PSA levels, especially seen in subjects with baseline PSA > 4 ng/ml. A statistically significant correlation between periodontal parameters and PSA levels after periodontal treatment.	Nabil and Bissada, 2015
Estemalik 2017	United States	Case series	14 CP/CPPS	Prostatic secretions	Etght of 14 subjects with CP/CPPS had no less than one oral pathogen in prostatic secretion specimens. *Parphyromonas gingivaiis*, *Treponema denticola*, and *Escherichia coli* were represented in prostatic secretions and dental plaques, respectively.	Estemalik et al., 2017
Boyapati 2018	India	Cross-sectional	100 subjects with comorbidity of CP/CPPS and periodontal disease	PSA	Poorer periodontal support tissues are more associated with moderate-to-severe prostate inflammation than with mild prostatitis. A significant positive correlation existed between moderate-to-severe prostatitis and periodontal parameters.	Boyapati et al., 2018
Fu 2021	China	Cohort study	5,510 subjects with periodontitis with therapy served as cohort,1, 5,510 subjects with periodontitis without therapy served as cohort,2, 5,510 subjects without diagnosis served as controls	–	A total of 68 of 5,510 subjects in cohort 1 ended up with prostatitis. A total of 70 of 5,510 subjects in cohort 2 ended up with prostatitis. A total of 34 of 5,510 subjects in controls ended up with prostatitis. Patients with chronic periodontitis, whether treated or not, are at a greater risk of developing prostatitis.	Fu et al., 2021
Alluri 2021	United States	Case series	A total of 30 subjects with whole-mount radical prostatectomy	Prostate tissue	(1) A periodontal pathogen, *F. nucleatum* subsp. *fusiform* strain ATCC 51190, is highly expressed in inflamed areas of the prostate gland.	Alluri et al., 2021
**Urinary microbiota**						
Nickel 2015	Canada	Case-control	110 CP/CPPS, 115 controls	VB1, VB2, and VB3	Different microbial composition was found in VB1 between CP/CPPS and controls.	Nickel et al., 2015
Shoskes 2016	United States	Case-control	25 CP/CPPS, 25 controls	VB2	Higher α-diversity was detected in the CP/CPPS group. Higher relative abundance of *Clostridia* and *Bacteroidia* were detected in the CP/CPPS group.	Shoskes et al., 2016a
Wu 2021	China	Case-control	33 CP/CPPS, 30 controls	Urethral secretions and EPS	The composition of the microbiota within the urethral secretions and EPS of the subjects was essentially the same. There was a significant difference in the α-diversity of the EPS microbiota between the CP/CPPS group and controls. Significant differences were found in 17 microorganisms in urethral secretions and 14 microorganisms in EPS in the CP/CPPS and control groups, respectively.	Wu et al., 2020
Liu 2023	China	Animal experiment	–	Fecal samples	EAP and/or depression can significantly alter the prostate microbial composition, change the relative abundance of prostate microbes, and induce prostate microbiota dysbiosis. Reductive acetyl coenzyme A pathway, L-lysine fermentation to acetate and butanoate, protein N-glycosylation, and purine nucleobases degradation I are associated with EAP/depression, and these pathways are regulated by *DCE29*, *Nocardioes*, *Helicobacter* and *Dorea*.	Liu et al., 2022
**Semen microbiota**						
Mandar 2017	Estonia	Case-control	21 CP/CPPS, 46 controls	Semen	Fewer health-supporting *lactobacilli* and higher species diversity were depicted in CP/CPPS subjects compared to their counterparts. *Firmicutes* (especially *lactobacilli*), *Proteobacteria*, *Bacteroidetes*, and *Actinobacteria* comprise the highest proportion of the seminal microbiome.	Mändar et al., 2017
Choi 2019	Korea	Case-control	17 CP/CPPS, 4 controls	Semen	Pyrosequencing revealed multiple bacterial genera in all samples, including an abundance of fastidious bacteria. *Corynebacterium*, *Pseudomonas*, *Sphingomonas*, *Staphylococcus*, and *Streptococcus* were frequently detected non-specifically in both the patient and control groups. *Achromobacter*, *Stenotrophomonas*, and *Brevibacillus* were more frequently found in CP/CPPS patients.	Choi et al., 2020

EAP, experimental autoimmune prostatitis; PSA, prostate-specific antigen; VB1, first void urine; VB2, midstream void urine; VB3, post-prostatic massage urine; EPS, expressed prostatic secretion.

**TABLE 2:** Novel microbiota-centric therapeutic strategies for chronic prostatitis/chronic pelvis pain syndrome (CP/CPPS).

Author (year)	Country	Microbe or substance	Main outcomes	References
**Certain microbes**				
Murphy 2017	United States	Non-pain-inducing (NPI)	Intraurethral instillation of NPI attenuates referred pelvic pain in EAP mice via lipoteichoic acid (LTA) in their cell walls rather than a mechanism dependent on strain colonization. *S. epidermidis* LTA (SELTA) specifically increased the expression of CD274 and CD273 cells (PDL1 and 2) in prostate tissue. IL-10 and T-cell (PD1- and CD25-expressing cells) immunomodulation play an important role in ameliorating tactile allodynia responses.	Murphy et al., 2017
Murphy 2018	United States	Non-pain-inducing (NPI)	NPI provides palliation and prevention of pathogen-induced inflammation and tactile allodynia by altering the immune response or inducing and maintaining an immune response in the already infected prostate gland. NPI instillation significantly reduced tactile allodynia but not CP1 colonization in CP1-infected mice. Prophylactic NPI instillation reduced CP1 colonization and tactile allodynia. NPI and CP1 strains do not inhibit each other’s growth or invasive ability, suggesting that alterations in bacterial assemblages may be host-induced rather than caused by any direct microbial interaction.	Murphy et al., 2018
**Substances**				
Konkol 2018	Finland	Galactoglucomannan (GGM)	Significant differences in the β-diversity of bacterial populations were detected between treatment groups. GGM extract treatment significantly reduced the abundance levels of *Odorobacteria* and *Clostridiaceae* and increased the levels of *Bacillus hominis* in CPI rats. GGM significantly increased serum levels of butyric and caproic acids and decreased serum levels of Lipopolysaccharide-binding protein (LBP).	Konkol et al., 2017
Konkol 2019	Finland	Galactoglucomannan (GGM)	Treatment with the GGM extract significantly reduced the abundance of *Odoribacter* and *Clostridiaceae* levels and increased the *Bacteroides uniformis* levels. The aforementioned imbalance in the microbiota may in turn induce reduced concentrations of butyric, valeric and hexanoic acids and elevated serum LBP, leading to the development of prostatitis in rats, which was reversed by GGM.	Konkol et al., 2019
Chen 2020	United States	Whey protein-derived early glycation products (EGPs)	Chronic oral exposure to EGPs mediates changes in the gut microbiota at six levels (phylum, class, etc.), reducing prostate inflammation and prolonging EAP mice’s lifespan. EGPs altered systemic immunity by modulating the content of immune cells and cytokines/chemokines, and changes in these immune parameters correlated significantly with the gut microbiota.	Chen Y. et al., 2020
Liu 2020	China	Poria cocos Polysaccharides (PPs)	PPs reduced weight, alleviated histological damage in inflamed prostate, and relieved CP/CPPS symptoms. PPs can alleviate CP/CPPS by altering the relative abundance of gut microbiota which is mainly represented by decreasing *Lachnospiraceae NK4A136 group*, *Lactobacillus*, and *uncultured bacterium f Erysipelotrichaceae* as well as increasing *Romboutsia*, and *uncultured bacterium f Desulfovibrionaceae*. PPs induce DNA methylation reprograming associated with hormone-related processes and may mediate changes in gut microbiota through these pathways. PPs reduce C-reactive protein (CRP) and pro-inflammatory cytokines (TNF-α and IL-β). PPs can alleviate CP/CPPS symptoms by modulating the production of testosterone (T), dihydrotestosterone (DTH), and estradiol (E2). PPs alleviate prostate inflammation by combating oxidative stress.	Liu et al., 2020
Liu 2021	China	Poria cocos Polysaccharides (PPs)	PPs reduced weight, alleviated histological damage in inflamed prostate, and relieved CP/CPPS symptoms. PPs significantly altered the composition of the gut microbiota. CNP-induced alterations in *Ruminococcaceae NK4A214 group*, *uncultured bacterium f Ruminococcaceae, Ruminiclostridium 9*, *Phascolarctobacterium*, *Coriobacteriaceae UCG-002*, and *Oribacterium* altered reversed by PPs, with *Ruminococcaceae NK4A214 group* being the central target of PPs for restoration of the gut microbiota. PPs inhibited the production of pro-inflammatory factors (TNF-α, IL-2, and IL-8) and androgens (testosterone and dihydrotestosterone).	Liu et al., 2021a
Yu 2022	China	Poria cocos Polysaccharides (PPs)	The PPs-mediated enrichment of *Parabacteroides*, *Fusicatenibacter*, and *Parasutterella* and the 7-ketodeoxycholic acid and haloperidol glucuronide metabolites produced by their fermentation of PPs played a key role in alleviating CP/CPPS. PPs-FM reduced pro-inflammatory factors (TNF-α, IL-6, and IL-1β) and oxidative stress products (MMP-9, iNOS, and COX-2) and had the effect of restoring serum sex hormones (DTH and E2). PPs-FM may influence the synthesis of sex hormones by regulating the expression of intestinal epithelial genes, which in turn modulates immunity and exerts inflammation-alleviating effects.	Yu et al., 2022
Liu 2024	China	Astaxanthin (AST)	Oral administration of AST alleviates CP/CPPS symptoms and suppresses prostate inflammatory responses by upregulating the relative abundance of *A. muciniphila*. Direct oral administration of *A. muciniphila* bacteria also improves CP/CPPS symptoms.	Liu et al., 2024
Tian 2024	China	Berberine hydrochloride	Berberine hydrochloride restored α- and β-diversity in the gut microbiota of EAP mice and altered the relative abundance of bacteria at the genus and species level. Berberine hydrochloride may mediate *Clostridium butyricum* binding to modulate the levels of SCFAs and thus alleviate symptoms.	Tian et al., 2024
Yu 2024	China	Eriocalyxin B (EriB)	EriB regulated the alpha and beta diversity of the gut microbiota. EriB alleviated prostatic inflammation and splenic M1/M2 macrophage ratio in mice. EriB-mediated changes in gut flora affect multiple metabolic pathways, prominently the metabolic pathway of vitamin D3, ultimately leading to macrophage polarization from the M1 to M2 phenotype.	Yu et al., 2024

NPI, non-pain-inducing; EAP, experimental autoimmune prostatitis; LTA, lipoteichoic acid; IL, interleukin; GGM, galactoglucomannan; LBP, lipopolysaccharide-binding protein; EGPs, whey protein-derived early glycation products; PPs, Poria cocos Polysaccharides; CPR, C-reactive protein; TNF-α, tumor necrosis factor-α; T, testosterone; DTH, dihydrotestosterone; E2, estradiol; PPs-FM, fermentation of PPs; MMP-9, matrix metalloproteinase-9; iNOS, inducible nitric oxide synthase; COX-2, cyclooxygenase-2; AST, astaxanthin; EriB, Eriocalyxin B.

## 2 Various microbiota of different regions and CP/CPPS

### 2.1 Gut microbiota and CP/CPPS

#### 2.1.1 Evidence of correlation between gut microbiota and CP/CPPS

The human gut harbors an extensive microbial ecosystem comprising over 10 trillion diverse microorganisms (Zmora et al., 2019). These coevolved microorganisms establish a symbiotic relationship with their host, thriving in the nutrient-dense intestinal environment. They perform crucial physiological functions while simultaneously being involved in disease processes by synthesizing a myriad of bioactive metabolites (Erny et al., 2021; Jugder et al., 2021). Applying the specific mode of producing small molecules, gut microbiota acts as a fundamental factor in the execution of protective, metabolic, and structural homeostasis (Adak and Khan, 2019; Gomaa, 2020). For instance, compelling evidence suggests that gut microbiota comprising its metabolic products significantly influence antitumor immune response, modulate immune checkpoint inhibitor efficacy, and impact cancer progression across multiple tumor types (Lu et al., 2022). In a like manner, gut microbiota affects prostatic pathophysiology by means of the above modes (Matsushita et al., 2021; Zhong et al., 2022).

Specific inflammatory conditions like CP/CPPS are also relevant to gut microbiota dysbiosis. In 2016, Shoskes et al. (2016b) conducted comparative analyses of gut microbiota composition between CP/CPPS patients and healthy controls. Their findings showed great interindividual variability in the gut microbiota and a lower α-diversity in CP/CPPS subjects, particularly in cases with symptom duration exceeding 48 months. In addition, several specific bacterial taxa were over- or under-represented, a case in point with the abundance of *Prevotella* (Shoskes et al., 2016b). Not limited to studies of the human microbiota, analogous results have been corroborated in animal models. Experimental autoimmune prostatitis (EAP) mouse model, established through subcutaneous injection of prostate antigens (PAg) along with adjuvants, remains the most widely utilized preclinical system for CP/CPPS research. The model recapitulates symptoms and immunologic features resembling human CP/CPPS (Breser et al., 2017). In 2019, Konkol et al. (2019) identified distinct microbial signatures in CP/CPPS rats, with five taxa showing increased abundance and four demonstrating decreased levels compared to healthy controls. Not coming solely but in pairs, Du et al. (2020) also noted comprehensive microbial alterations across all taxonomic levels (phylum, class, order, family, genus, and species) in the EAP models. Supporting these findings, subsequent investigations by the same team identified specific microbial alterations across multiple taxonomic hierarchies. It warrants mentioning that the EAP group showed a decrease in all nine bacterial groups at the genus level, except for *Lactobacillus*, which increased (Du et al., 2022), enhancing the synthesis of short-chain fatty acids (SCFAs) (Markowiak-Kopeć and Śliewska, 2020). Parallel research revealed distinct β-diversity patterns and identified three enriched and seven depleted bacterial taxa in CP/CPPS cohorts (Wang S. et al., 2023). Taken together, the findings above provide a compelling association between gut microbiota alterations and CP/CPPS pathogenesis. Interestingly, recurrent observation in α-diversity alterations might suggest its potential utility as a diagnostic biomarker for CP/CPPS. Whereas, such abnormal α-diversity may also reflect confounding factors including prolonged antibiotic exposure, aging, or systemic physiological changes (Haak et al., 2019; Odamaki et al., 2016). Furthermore, specific microbial alterations might represent either protective or etiological features. *Prevotella*, a dietary fiber-responsive bacterial genus that predominates in individuals consuming plant-rich diets, has been implicated in energy metabolism optimization and anti-inflammatory processes. Its established health-promoting properties and observed depletion in CP/CPPS patients suggest its potential dual role as both a diagnostic biomarker and therapeutic target for this condition (Ley, 2016).

Of note, mounting lines of evidence enlightened that there are evident interrelationships between gut microbiota composition and psychological status in CP/CPPS patients (Huang et al., 2019; Kelly et al., 2019). In clinical diagnosis and treatment, psychological dysfunction may be concomitant with physical discomfort in patients suffering from CP/CPPS. Up to 78% of patients showed comorbid depression when compared with the general male population (Egan and Krieger, 1994). Analogous to CP/CPPS, depression exhibits complex etiology, and while the causal relationship remains undefined, their parallel progression is elucidated (Stamatiou et al., 2023). It has been verified, at least in animal models, that prostate inflammation induces depressive symptomatic behavior and cognitive declines (Šutulović et al., 2023). Extensive gut-brain axis-related studies have emerged to provide supporting evidence for the development of depression, and one of the contributors appears to be tied to the gut microbiota (Stower, 2019). Given the fact that altered gut microbiota exists in patients with CP/CPPS, researchers have actively explored the hypothesis that CP/CPPS primarily mediates the gut microbiota leading to depression. Concentrating on this area, Du and his colleagues utilizing the EAP model successively conducted animal experiments that showed much support for such theory that CP/CPPS may lead to the alteration of gut microbiota and induce depressive symptoms (Du et al., 2020; Du et al., 2022).

#### 2.1.2 Possible mechanisms

The underlying mechanism of CP/CPPS remains incompletely characterized. One of the mechanisms, by which the gut microbiota exerts their protective effects, is to occupy the intestine surfaces and maintain intestinal barrier integrity to block the invasion of foreign pathogenic microbes (Karczewski et al., 2010). This mechanism provides substantial support for the hypothesis that CP/CPPS pathogenesis may involve compromised microbial barrier function, permitting pathogen translocation (Song et al., 2023).

**FIGURE 2:**
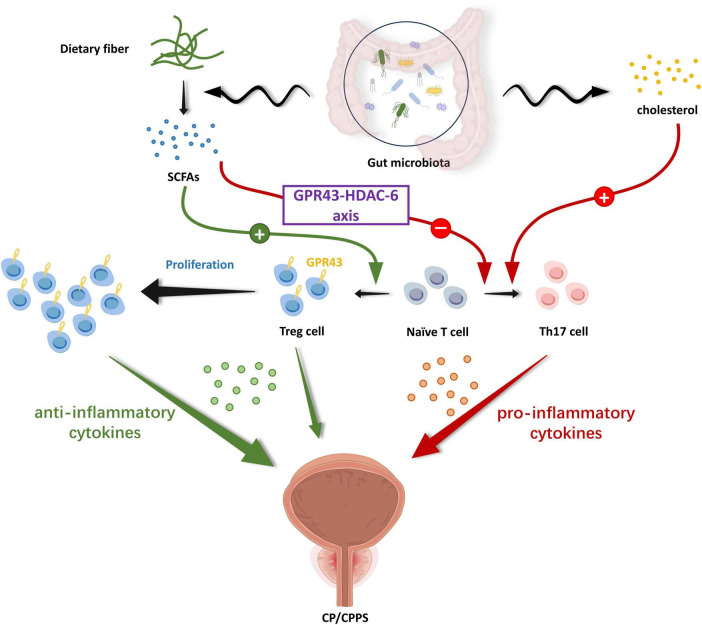
The gut-prostate axis: immune mechanism. SCFAs produced by gut microbiota decomposing dietary fibers in food possess the ability to regulate the proliferation and differentiation of immune cells via the corresponding G protein-coupled receptor pathway. Gut microbiota-derived SCFAs propionate can promote the differentiation of initial T cells to Treg cells and inhibit their differentiation to Th17 cells via the GPR43-HDAC-6 axis, with the former secreting anti-inflammatory cytokines and the latter secreting pro-inflammatory cytokines. Furthermore, gut microbiota up-regulate Th17 cell ratios and their responses through microbial-associated cholesterol metabolism pathways, exacerbating the inflammatory response. The relative balance of Th17/Treg is critical in the inflammatory environment of the prostate. This immune mechanism by which the gut microbiota and its metabolites drive host immune regulation has further deepened researchers’ understanding of the gut-prostate axis. (SCFAs, short chain fat acids; GPR, G protein-coupled receptor; HDAC, histone deacetylase).

Aside from the direct pathway, CP/CPPS bears a relation to disrupting the homeostasis of gut microbiota and the secondary changes it engenders in metabolites resulting from compositional changes (Konkol et al., 2019). In the study of Liu et al. (2021b), they used multi-omics analysis to explore the alteration in the composition of gut microbiota, gene expression, and DNA methylation in the CP/CPPS rat model. Their analysis displayed 185 differentially expressed genes in the intestinal epithelium and identified 73,232 differentially methylated sites (DMSs). In another study by Du et al. (2022), a significant imbalance in the ratio of Th17 cells to Treg cells in the EAP group drew extensive concerns from researchers. Thus, they proposed that the propionate might modulate T cell differentiation through the GPR43-HDAC6 axis, restoring the balance of Th17 and Treg cell ratios. Their updated findings indicated alcohol-mediated gut microbiota alterations stimulate Th17 differentiation and response via the cholesterol biosynthesis metabolic pathway, which ultimately exacerbated CP/CPPS symptoms. The cholesterol biosynthesis regulator SREBP2 played an integral function in this pathway (Du et al., 2024). Taking all results into account, the changes in gut microbiota might alter epithelial gene expression, successively modulating the immunocytes such as Th17/Treg cells balance and their respective pro-/anti-inflammatory cytokine profiles, thus ultimately exerting systemic effects (Wang J. et al., 2023). In addition, the outstanding performance of propionate captured our attention. SCFAs, particularly propionate, domain a leading part of metabolites generated by the gut microbiota through colonic fermentation of undigested carbohydrates, with well-established roles in inflammatory regulation (Cummings et al., 1987; Hamer et al., 2008; Trompette et al., 2014). They exert their effects in a manner of binding to the corresponding G protein-coupled receptors (GPR) such as GPR43 or by interacting with histone deacetylase (HDACs), initiating downstream signaling cascades thus resulting in the corresponding cellular effects (Rooks and Garrett, 2016; Sivaprakasam et al., 2016). Collectively, these findings position gut microbiota as a crucial agent in driving the regulation of host immunity, primarily through microbial metabolites including SCFAs (Schluter et al., 2020; Smith et al., 2013). These mechanistic insights into gut microbiota affecting CP/CPPS have led to the proposal of a gut-prostate axis, which resembles the gut-brain axis, gut-lung axis, and gut-liver axis, etc., (Cryan et al., 2019; Du et al., 2022). The gut-prostate axis builds bidirectional communication between intestinal and prostatic systems which paved the way for researchers to increasingly recognize various domains (Figure 2), but it still warrants further investigation.

### 2.2 Oral microbiota and CP/CPPS

#### 2.2.1 Evidence of correlation between oral microbiota and CP/CPPS

A majority of investigators have predominantly attempted to explain the causative efficacy in CP/CPPS of the microbiota which is located in the urogenital system, yet they seldom notice that oral microbiota has unique interest in CP/CPPS. Existing research suggests that oral health status is firmly associated with the development of several systemic diseases (Babaev et al., 2017; Suresh et al., 2016). Accumulating oral microbiota-centric research has substantiated this phenomenon, with a wealth of evidence that oral microbiota may act as suspicious etiologic agents in the onset of prostatic diseases (Boland et al., 2013; Wu et al., 2019). More critically, a couple of important studies have also exemplified both direct and indirect mechanistic links between the oral microbiota and CP/CPPS.

To start with, several studies have provided indirect evidence of the association of oral microbes with CP/CPPS. Elevated serum prostate-specific antigen (PSA), a well-established prostate cancer biomarker, showed a statistical association with CP/CPPS (Nadler et al., 2006). It is indicated that prostatic inflammation contributes greatly to elevating serum PSA concentrations (Nickel et al., 1999; Yaman et al., 2003). Zooming into previous epidemiological studies, PSA level also seems to be statistically associated with periodontal disease (Boyapati et al., 2018; Joshi et al., 2010). Merging epidemiological studies have shown an indirect correlation between CP/CPPS and periodontal disease through the intermediary of PSA. Interestingly, periodontal treatment has been shown to reduce serum PSA level and alleviate CP/CPPS symptoms (Nabil and Bissada, 2015). Herein, there may be potential PSA-mediated connections between periodontal health and CP/CPPS.

Beyond indirect associations, some evidence also objectively suggests a relatively direct potential relationship between CP/CPPS and oral microbiota. Initial small-scale case analyses isolated exact oral pathogens from EPS of comorbid patients with periodontal disease and CP/CPPS. The investigators found that a total of 8 of 14 (57.1%) subjects had no less than one oral pathogen in their samples (Estemalik et al., 2017). In another cross-sectional study by Boyapati et al. (2018), they found the mean clinical attachment level, a standard parameter that reflects the extent of periodontal tissue destruction, notably increasing in moderate-to-severe CP/CPPS patients in spite of mild CP/CPPS patients (Sanz et al., 2020). A cohort study selected periodontitis patients who were excluded from prior CP/CPPS diagnosis into two cohorts of patients with and without periodontitis treatment, and a matched control cohort without periodontitis was set. At the endpoint, the characteristic patterned increased CP/CPPS susceptibility among periodontitis patients, regardless of treatment status (Fu et al., 2021). Alluri et al. (2021) isolated *Fusobacterium nucleatum* subsp. *fusiform* strain ATCC 51190, a periodontal pathogen, in histologically abnormal prostate tissues, suggesting its potential role in linking periodontal disease and prostatic inflammation. As noted above, we can hypothesize that there is a correlation between oral microbiota and CP/CPPS. However, cross-sectional studies are limited to probing causality, and further randomized controlled trials (RCTs) and experimental validations remain necessary to elucidate oral microbiota’s role in CP/CPPS pathogenesis.

#### 2.2.2 Possible mechanisms

**FIGURE 3:**
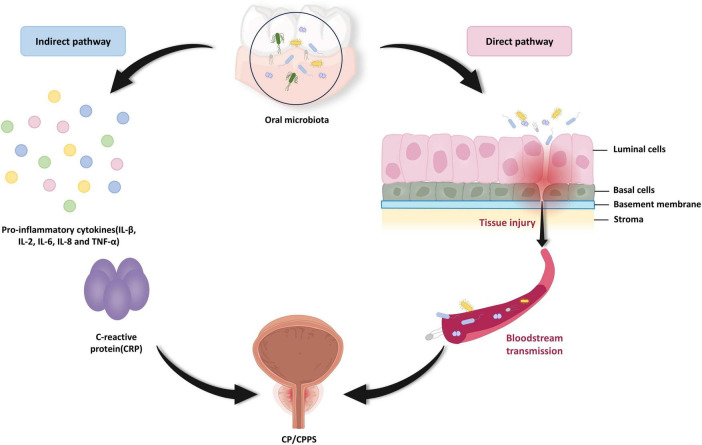
The possible mechanisms of oral microbiota-induced chronic prostatitis/chronic pelvis pain syndrome (CP/CPPS). Oral pathogens may reach the prostate via hematogenous spread, thereby directly injuring prostate tissue. In addition to this, oral pathogens may mediate the production and dissemination of pro-inflammatory factors, such as IL-1β, IL-2, IL-6, IL-8, TNF-α, and CRP that indirectly trigger CP/CPPS onset. This hypothesis of an indirect pathogenic pathway provides an argument for systemic immune disorders to lead to CP/CPPS. (IL, interleukin; TNF-α, tumor necrosis factor-α; CRP, C-reactive protein).

The precise mechanism through which oral microbiota affects CP/CPPS pathogenesis remains elusive, with the available evidence principally supporting two primary direct pathways, microbial translocation and indirect systemic inflammation. For the direct pathway, previously mentioned results suggest the hypothesis that hematogenous dissemination of oral microorganisms to the prostate triggers histological changes that contribute to the occurrence of CP/CPPS (Alluri et al., 2021; Estemalik et al., 2017). For the indirect pathway, increasingly circulating levels of periodontal-derived pro-inflammatory factors, including interleukins (IL-1β, IL-2, IL-6, IL-8), tumor necrosis factor (TNF-α), and C-reactive protein (CRP), may act as indirect agents in the onset of CP/CPPS (Boyapati et al., 2018; Hegde and Awan, 2019). These locally produced, periodontitis-derived inflammatory mediators can trigger a systemic inflammatory response through the circulatory system, which in turn causes or exacerbates CP/CPPS (Figure 3).

### 2.3 Urinary microbiota and CP/CPPS

In an extraordinarily long period, the longstanding paradigm of urinary sterility has dominated throughout the entire research process owing to conventional culture methods. However, next-generation sequencing (NGS) has revolutionized this concept, revealing complex microbial ecosystems within the urinary tract (Wolfe et al., 2012). Given the anatomical proximity and shared urothelium, urinary microbiota might be of particular interest to prostatic diseases (Porter et al., 2018). Accordingly, a tremendous amount of energy was invested into the domain of how urinary microbiota affects human health with all sorts of evidence denoting that urinary microbiota plays a role in urinary diseases (Shrestha et al., 2018).

Nickel et al. (2015) yielded the first glimpse into urinary microbiota and CP/CPPS and, identified an excess of *Burkholderia cenocepacia* in first-void urine (VB1) specimens from patients. Correspondingly, a study by Shoskes et al. (2016a) subsequently demonstrated increased *Clostridia* and *Bacteroidia* and elevated α-diversity in CP/CPPS patient samples. Their findings revealed a statistical correlation between the severity of patient symptoms and the composition of the urinary microbiota, in addition to a significant increase in the bacterial diversity of the urinary microbiota as the symptom score increased (Shoskes et al., 2016a). Recently, Wu et al. (2020) employed high-throughput NGS to demonstrate a similarity in the composition of microbial communities between urethral secretions and EPS samples in CP/CPPS patients and healthy populations, respectively. Meanwhile, seventeen bacterial species were differentially represented in the urethral secretions in cases versus controls, though no typical pathogens were detected in CP/CPPS patients. As noted above, *B. cenocepacia*, an opportunistic human pathogen, was described as potentially involved in the etiology of CP/CPPS (Organ et al., 2010; Schwager et al., 2013). The abnormally richer α-diversity and the specific class-level alterations may represent promising clinical diagnostic biomarkers. To clarify, the discovery of Wu et al. implies that non-cellular microbes in CP/CPPS patients provide new insights into CP/CPPS etiology and mechanisms.

To verify the infection hypothesis of CP/CPPS-associated chronic pain is clinically relevant to infection, Rudick et al. (2011) inoculated murine models with CP1, an atypical isolate distinct obtained from EPS and VB3 of a CP/CPPS patient. They showed CP1’s ability to induce and maintain chronic pelvic pain in murine models. Aside from this specific Gram-negative bacterial isolate, the same laboratory subsequently demonstrated that multiple Gram-positive patient-derived bacteria were up to trigger CP/CPPS-like symptoms in murine models (Murphy et al., 2019). Its induction mechanism appears to involve STAT6-mediated regulation of IL-4 and IL-13 secretion (Bell-Cohn et al., 2019). These findings establish a foundation for developing more representative animal models and may address gaps in the etiology of CP/CPPS.

As is presented psychiatric comorbidities (particularly depression and somatization) were found to have negative impacts on CP/CPPS symptom severity, and some adequately powered studies were designed concerning prostate microbiota to address this clinical challenge (Koh et al., 2014). Liu et al. (2022) demonstrated a multitude of prostate microbiota composition was aberrant in their assay of EAP/depression mice. In tandem, several bacteria-related metabolic pathways were confirmed to be correlated with both EAP and depression. These findings provide novel therapeutic targets for addressing CP/CPPS-depression comorbidity.

### 2.4 Seminal microbiota and CP/CPPS

One of the classifications of CP/CPPS is the presence of leukocytes and bacteria in the EPS, the composition of which provides insights into prostate health status (Krieger et al., 1999). Song et al. (2024) detected characteristic microbes in the EPS from CP/CPPS patients, with significant compositional changes in patients who underwent effective low-intensity pulsed ultrasound therapy. Taking credit for the constituent of semen and EPS shares high similarity, semen analysis serves as an alternative diagnostic approach when EPS collection is challenging (Motrich et al., 2018). However, this alternative has shortcomings, as semen-derived microbes and inflammatory markers may originate from non-prostatic sources, which can lead to clinical diagnosis bias (Kermes et al., 2003). Nonetheless, some microbes are significantly enriched in male semen with urinary diseases, and quantitative microbial parameters also show salient correlations with inflammation (Korrovits et al., 2006).

Mändar et al. (2017) analyzed the semen microbiota of CP/CPPS patients and healthy controls by applying 16S rRNA gene sequencing. They found that fewer health-supporting *Lactobacillus* and higher species diversity were depicted in CP/CPPS subjects compared to their counterparts. The predominant phyla detected in semen samples are *Firmicutes*, *Bacteroidetes*, *Proteobacteria*, and *Actinobacteria*. In another pilot study, Choi et al. (2020) displayed the discrepancy that *Achromobacter*, *Stenotrophomonas*, *Brevibacillus* were more commonly present in the semen of CP/CPPS patients. Both of the results showed the association between semen microbiota and CP/CPPS, suggesting the possible protective effects of *Lactobacillus*.

## 3 Novel therapeutic strategies for CP/CPPS by targeting microbiota

As the etiology of CP/CPPS is undetermined, empirical antibiotic therapy remains a common clinical approach (Rees et al., 2015). Abusing antibiotics entails several undue toxicities, which can’t be underestimated (Singh et al., 2017). Promisingly, several substances that can modulate microbial abundance and specific commensal isolates have shown initial symptom-relieving efficacy in preclinical studies.

### 3.1 Certain microbes exerted symptom-relieving efficacy

Some studies in recent years have shown that bacteria isolated from healthy populations appear to ameliorate symptoms of CP/CPPS. The non-pain-inducing (NPI) strain of *Staphylococcus epidermidis*, isolated from the prostatic secretions, has been detected to lessen the symptoms in murine models with CP/CPPS and increase CD4^+^IL-17A^+^T-cell populations (Murphy et al., 2017), known to be salient in disease orchestration and pelvic tactile allodynia development (Murphy et al., 2015). Further on, the team also found that prophylactic instillation of NPI palliated colonization, pain response, and immune activation of urinary pathogens (Murphy et al., 2018). The discoveries highlight the therapeutic potential of human commensal bacteria and warrant further investigation into their clinical applications for CP/CPPS management.

### 3.2 Substances exerted symptom-relieving efficacy by targeting microbiota

Meanwhile, some compounds are competent to regulate the relative abundance of microbes upstream, thereby improving the symptoms in animal models.

#### 3.2.1 Glycated proteins (EGPs)

Chen Y. et al. (2020) administered whey protein-derived early glycation products (EGPs) at 600 mg/kg/day to non-obese diabetic (NOD) mice with concurrent spontaneous autoimmune prostatitis. After undergoing 6 months of rearing, EGP-treated mice exhibited significantly improved survival rates compared to controls. Moreover, attenuated prostate inflammation, decreased splenic M1 macrophage and lymphocyte populations, as well as increased systemic anti-inflammatory factor levels were exhibited in the group with EGPs. Changes in the relative abundance of gut microbiota and increased *Bacteroides acidifaciens* counts paralleled the alleviation of inflammation, perhaps correlating the intrinsic mechanism of EGP in regulating the systemic immune status in a manner.

#### 3.2.2 Galactoglucomannan (GGM)

In Konkol et al. (2017) study, galactoglucomannan (GGM) was clarified to possess the ability to improve lower urinary tract symptoms (LUTS) associated with chronic prostatic inflammation in rat models. In subsequent experiments, they established a non-bacterial prostatitis model with subcutaneous testosterone and 17β-estradiol hormone pellets on adult male Wister rats, administering water containing 2% GGM orally for feeding whilst the control group was given tap water. Following 5 weeks of treatment, GGM normalized prostate inflammation-induced elevations in *Odoribacter* and *Clostridiaceae* levels while increasing *Bacteroides uniformis* abundance. In addition, GGM was demonstrated to significantly increase the serum levels of butyric acid and caproic acid while lipopolysaccharide binding protein (LBP) concentrations, thereby contributing to relieving the LUTS of prostatitis (Konkol et al., 2019).

#### 3.2.3 Poria cocos Polysaccharides (PPs)

Liu et al. (2020, 2021a) found that Poria cocos Polysaccharides (PPs) pre-treated with 250 mg/kg per day by gavage for 7 days alleviated inflammation in a non-bacterial prostatitis model induced by intraperitoneal injections of 1% λ-carrageenan in male Sprague Dawley (SD) rats, with gut microbiota modulation, particularly targeting the *Ruminococcaceae NK4A214* group to ameliorate symptom. In addition to the blank control and the untreated group, the researchers installed a finasteride-treated group, the outcome showed that PPs could target different genera compared to the finasteride group, thus exerting therapeutic effects. Research thereafter clarified that PPs may modulate several key bacteria and metabolic outputs, including 7-keto-deoxycholic acid and haloperidol glucuronide, resulting in palliative effects (Yu et al., 2022).

#### 3.2.4 Astaxanthin (AST)

Astaxanthin (AST), a compound with potent antioxidant, anti-inflammatory, and immunomodulatory properties (Chen Z. et al., 2020), has recently been the subject of related studies that have demonstrated its value in upregulating the relative abundance of *Akkermansia muciniphila*, which in turn ameliorating CP/CPPS symptoms. NOD mice were stratified into four groups: control, EAP, EAP + AST 50 mg/kg group, and EAP + AST 100 mg/kg group. All mice excluding the control group received oral AST administration every other day for 6 weeks starting from the first subcutaneous immunization until the end of the experiment, following a second immunization to obtain the EAP model at the fourth week. Feces from CP/CPPS patients exhibited a reduction of *A. muciniphila* in the gut microbiota compared to the healthy population the same as in EAP mice versus the normal mice, whereas AST treatment restored *A. muciniphila* levels in EAP models. *A. muciniphila* transplantation to EAP mice mirrored similar benefits of AST intervention in elevating the concentration of SCFAs, especially acetic acid which ultimately eases prostate inflammation (Liu et al., 2024).

#### 3.2.5 Berberine hydrochloride

Berberine hydrochloride is known to work in the therapeutic area across various diseases. In EAP models, daily administration of berberine hydrochloride 200 mg/kg for 4 weeks restored the α- and β-diversity of the gut microbiota, reduced prostate inflammation, and mitigated alterations in the relative abundance of the bacterial species at the genus and species level. Strikingly, *Clostridium butyricum* increased SCFA production in the course that berberine hydrochloride regulates the intestinal microbiota, representing a candidate target of the berberine hydrochloride effect. Fecal microbiome transplantation (FMT) based on pseudo-germ-free mice (PGFR) revealed that recipient mice receiving microbiota from berberine hydrochloride-treated EAP donors exhibited more severe inflammatory reactions than those receiving untreated EAP microbiota, reaffirming the mitigating effect of berberine hydrochloride (Tian et al., 2024).

#### 3.2.6 Eriocalyxin B (EriB)

In the work of Yu et al. (2024), prostate inflammation was significantly alleviated in EAP mice receiving daily oral gavage of EriB 10 mg/kg for 14 consecutive days, as well as altered gut microbiota α- and β-diversity in EAP mice compared to untreated controls. Such changes in microbiota composition altered the metabolic pathway of vitamin D3, promoting macrophage polarization from pro-inflammatory M1 to anti-inflammatory M2. Subsequent FMT in PGFR confirmed these findings, showing reduced M1/M2 ratios in recipients of EriB-treated donor microbiota compared to the EAP group without EriB, further substantiating the above mechanism.

While promising, the mutual deficiency of these studies is the boundedness of murine models. Their pharmaceutical validity and safety for human application are unverified, necessitating human-appropriate formulations and large-scale clinical trials. Nevertheless, these compounds represent promising candidates to help develop rational alternative treatment scheme regimens of CP/CPPS at this stage.

## 4 Conclusion and future perspectives

Overwhelming evidence demonstrates that alterations in human microbiota are intimately associated with CP/CPPS, which raises informed conjectures about the pathogenesis of this clinically intractable dilemma. With respect to the advances in sequencing technologies, microbiota and their metabolic products were proven to play an important role in CP/CPPS pathophysiology. The utilization of gut microbiota and microbiota-related metabolites as novel promising biomarkers for CP/CPPS prediction and diagnosis, as well as targets for disease management, has shown immense research interest. Future research directions will focus on microbiota-targeted interventions such as probiotics, prebiotics, and fecal microbiota transplantation, offering safer and more effective clinical alternative treatment schemes to current therapies with fewer adverse effects.
